# Development of a CT-less SPECT Acquisition Protocol for Kidney Dosimetry in ^177^Lu-PSMA-617 Radioligand Therapy

**DOI:** 10.1007/s11307-025-01998-2

**Published:** 2025-03-20

**Authors:** Christian Happel, Larissa Völler, Benjamin Bockisch, Daniel Groener, Britta Leonhäuser, Frank Grünwald, Amir Sabet

**Affiliations:** 1 Department of Nuclear Medicine, Clinic for Radiology and Nuclear Medicine, Goethe University Frankfurt, University Hospital Frankfurt, Theodor Stern Kai 7, D-60590 Frankfurt, Germany; 2 Partner Site Frankfurt/Mainz and German Cancer Research Center (DKFZ), German Cancer Consortium (DKTK), Heidelberg, Germany

**Keywords:** Kidney dosimetry, Radioligand therapy, ^177^Lu-PSMA

## Abstract

**Purpose:**

Targeted radioligand therapy of metastatic castration-resistant prostate cancer (mCRPC) with ^177^Lu-PSMA (RLT) requires sufficient dose monitoring of the kidneys. Currently, dosimetry using SPECT/CT-imaging is the most preferred method. However, SPECT/CT is a time-consuming procedure and comprises additional radiation exposure to the patient. Moreover, not every therapeutic nuclear medicine facility has access to SPECT/CT. Therefore, the aim of this study was to develop a new procedure of kidney dosimetry without the use of SPECT/CT and evaluate this method in a large cohort of patients with mCRPC undergoing RLT.

**Procedures:**

A dedicated torso phantom with kidneys filled with a solution of ^177^Lu-PSMA was used for quantitative calibration of a SPECT-camera. The calculated sensitivity was adapted according to the individual attenuation of the patient in four directions from the kidney surface to the body surface (ventral, dorsal, left and right) obtained from a previously performed CT. A total of 196 patients undergoing 926 cycles of ^177^Lu-PSMA therapy were retrospectively analyzed. Abdominal SPECT was performed 24, 48 and 72 h after administration of ^177^Lu-PSMA including scatter and dead-time correction in every patient. Kidney dose was calculated using an individual attenuation-based procedure and compared to values from international literature.

**Results:**

Volumes of interest of the kidneys were drawn in the three sequential SPECT-images to calculate intra-renal effective half-life. Absolute quantification of activity in the kidneys was accomplished obtaining a patient individual sensitivity based on the individual attenuation in the patient. Kidney dose was then calculated applying a bi-exponential time activity curve in Microsoft EXCEL. Mean kidney dose per administered activity was 0.54 (± 0.26) Gy/GBq.

**Conclusions:**

With the presented procedure a reliable kidney dosimetry is possible without the use of SPECT/CT. Facilities without SPECT/CT are therefore able to perform an adequate kidney dosimetry without additional radiation exposure for the patient.

## Introduction

Most common therapeutic procedure in nuclear medicine is the well-established radioiodine-131 therapy [1–3], yet the most researched therapeutic option is ^177^Lu-PSMA radioligand therapy (RLT) in metastatic castration-resistant prostate cancer (mCRPC) [4–11]. While intra-therapeutic dosimetry has been a standardized procedure in radioiodine therapy for decades, ^177^Lu-PSMA dosimetry is currently under constant development and not yet completely unified in generally accepted regulations [1–3, 12–16]. Prostate-specific membrane antigen (PSMA) can be labelled with different radionuclides and therefore be used for diagnostic and therapeutic purposes respectively [17]. The diagnostic tracer complex ^68^Ga-PSMA is used for PET/CT imaging to evaluate and quantify expression of PSMA [4, 18–20], whereas ^177^Lu-PSMA is applied for therapeutic interventions. This close combination of individualized diagnostic and treatment is commonly known as “theranostics” [21, 22]. The implementation of RLT does not yet necessarily prescribe dosimetric monitoring of the activity accumulation and elimination of the tracer in the body [15, 23]. In addition to cancer tissue, PSMA is amongst others physiologically expressed in the proximal tubules of the kidneys [24–26]. ^177^Lu-PSMA is administered intravenously and is excreted by the kidneys. Therefore, the renal function of each patient should be observed prior to RLT [27]. Nephrotoxicity of RLT has not been described as a frequent occurrence so far, but this might be due to strict dose and cycle limitations and the current absence of long-term experience [24, 28, 29]. Therefore, renal function may only be slightly impaired at the beginning of treatment [15, 27]. The dose limit for the kidneys originally described in radiation-oncology is defined to be 23 Gy [30]. However, since the dose is administered with a much lower dose rate in therapy with intravenously administered radioactive isotopes than in external radiation therapy, accumulated kidney doses of up to 28 – 40 Gy are acceptable in patients depending on other potential risk factors [15, 31].

Since there is no established standard for dosimetry of organs at risk in ^177^Lu-PSMA RLT, several approaches for example determination of the biological kinetics of administered ^177^Lu-activity using sequential scintigraphic whole-body images, laboratory parameters or SPECT/CT imaging have been discussed in the literature [26, 32–37]. However, a combination of SPECT and SPECT/CT images is currently the preferred method to calculate the individually administered radiation dose to the kidneys [38–41]. One of the major advantages of SPECT/CT imaging, beside an improved anatomical localization, organ contouring and organ activity quantification, is the option to perform attenuation correction, which is possible with the integrated CT-component. However, SPECT/CT may not be accessible ubiquitously. To enable the determination of a quantitative time activity curve in the kidney without the use of a SPECT/CT, a new dosimetric method depending on individual attenuation in the patient obtained from previously performed CT images was developed. This method is based only on sequential intra-therapeutic SPECT images and a previously performed CT. Applying this method, the overlay of organs and tissue in the kidney region can be excluded to determine the time activity curve within the kidneys quantitatively and therefore to calculate the absolute kidney dose without the use of a SPECT/CT.

Therefore, the aim of the current study was to describe and evaluate the developed method in a large cohort of patients. The process, the results and the limitations of this proposed attenuation-based dosimetry shall be presented and discussed in comparison to the established dosimetric method using SPECT/CT data, control measurements using a dedicated fillable torso phantom, quantitative evaluation using a designated software, quantification using planar scintigraphy as well as data from current literature. Aim of the proposed method is to provide a route to substantially improve quantitative accuracy of CT-less gamma camera acquisition in kidney dosimetry for ^177^Lu-PSMA-RLT.

## Material and Methods

In this retrospective study, 196 patients with mCRPC undergoing a total of 926 ^177^Lu-PSMA treatment cycles were evaluated. The mean age of the patients was 72.9 ± 7.5 years at initial administration (Tab. 1). The study was approved by the local ethics committee. RLT was indicated according to current procedure guidelines in an interdisciplinary tumor conference [13, 15]. A sufficient PSMA expression was confirmed in advance and between the treatment cycles by ^68^ Ga-PSMA PET/CT. The administered ^177^Lu activity and the number of treatment cycles were patient-specific and dependent on tumor burden, clinical condition, renal and bone marrow function and previously conducted treatment cycles. Labelling was performed directly prior to administration. The radionuclide ^177^Lu was obtained un-complexed from ITM (Isotopen Technologien München AG) and the ligand PSMA-617 from ABX (advanced biochemical compounds GmbH). Patients were hospitalized for at least 72 h after administration [16]. ^177^Lu-PSMA was administered intravenously in a solution of sodium chloride. The mean administered activity was 7.15 (± 1.5) GBq per treatment cycle (Tab. 1). During the three days after administration (24 h, 48 h and 72 h) daily sequential abdominal SPECT acquisitions were performed with a scintillation camera equipped with a $5/8$" NaI(Tl) scintillation crystal and a medium energy collimator (MEAP) (Mediso AnyScan; 64 steps, 15 s/step, energy window: 208 keV ± 10%; triple window scatter correction). Volumes of interest (VOI) of the left and the right kidney were drawn in the three sequential SPECT to calculate intra-renal effective half-life applying a bi-exponential fit in Microsoft EXCEL. The analysis of the SPECT images was performed with the designated software InterView XP (Mediso Imaging Systems; V3.06.007.0001).

**Table 1:** Characteristics of the study population

Patients	Cycles	Mean age [a]	Mean body weight [kg]	Mean administered activity [GBq]
196	926	72.9 ± 7.5	82.4 ± 14.9	7.15 ± 1.5

For absolute quantification of activity in the patients’ kidneys, the tomographic sensitivity of a ^177^Lu filled kidney phantom was used. The kidney phantom (volume 109 ml) was filled with a solution of ^177^Lu-PSMA-617 and sodium chloride. A SPECT examination of the kidney phantom was performed (Mediso AnyScan; 64 steps, 15 s/step, energy window: 208 keV ± 10%; triple window scatter correction) and revealed a decay, scatter and background corrected tomographic sensitivity of 10.2 cps/MBq. The tomographic sensitivity cannot be used for patient measurement due to individual attenuation by overlapping organs, bones and tissue in the patient. Therefore, individual tomographic sensitivity was calculated for each patient by measuring the distance from the surface of the kidneys to the surface of the patient body in four directions (ventral, dorsal, right and left) on CT images of the previously performed ^68^Ga-PSMA PET/CT. The attenuation coefficient of ^177^Lu and water was calculated to be 0.108 cm^−1^. It was calculated by sequential decay corrected measurements of a ^177^Lu filled syringe shielded with five layers of acrylic glass with different thicknesses (3 cm—20 cm) and a density of 1.2 g/cm^3^. The resulting attenuation coefficient of 0.130 cm^−1^ was adapted to the density of water (1 g/cm^3^) to be 0.108 cm^−1^. Using this attenuation coefficient, the individual attenuation of each patient in the four directions can be calculated using the law of attenuation. The mean density in the patient is individually dependent on body composition and body mass index and was therefore assumed to be 1 g/cm^3^ [42]. A mean attenuation-based sensitivity determined on those measurements was calculated individually for every single patient. Using this individual tomographic sensitivity, the absolute quantification of activity in the kidneys is possible (Table 1).

**Fig. 1:**
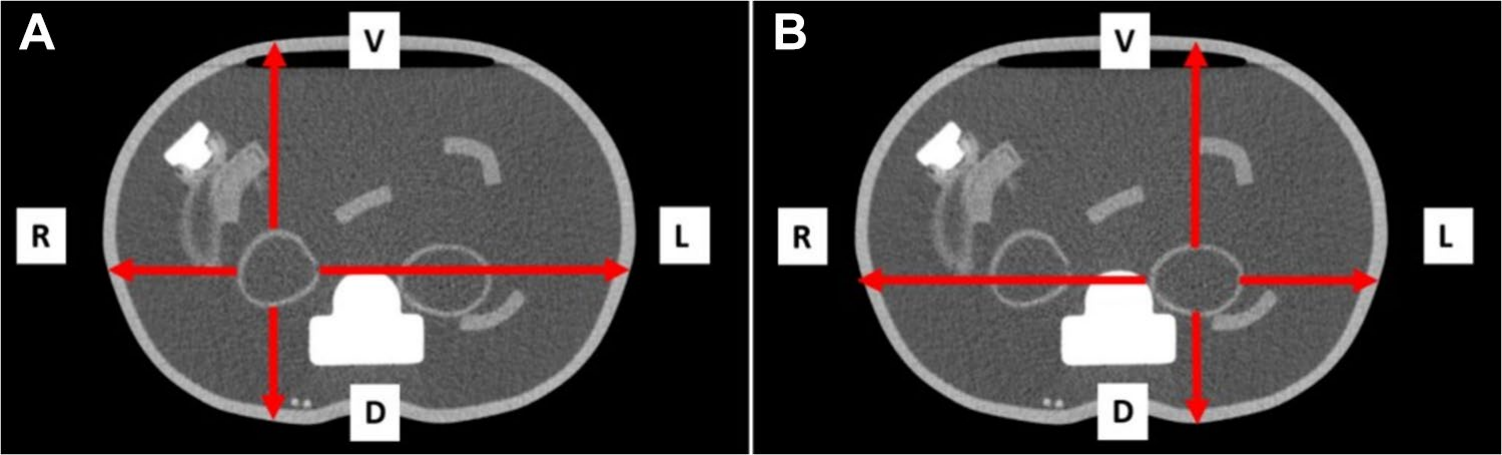
Transaxial CT-view of the torso phantom with measurement of the distances from the right kidney (**A**) and the left kidney (**B**) to the corresponding phantom surface

As a proof of concept, the known absolute activity-concentration in the kidneys of a fillable torso phantom was determined using the method here described. The distances from the surfaces of both kidneys to the ventral, dorsal, right and left surface of the torso phantom were obtained by CT imaging of the phantom prior to filling. The linear attenuation coefficient of ^177^Lu and water (0.108 cm^−1^) was used to calculate the individual attenuation of the filled torso phantom. Figure 1 shows a transversal slice of the torso phantom with the distances for the right (A) and the left (B) kidney (Fig. 1). The measured distances are shown in (Table 2). Using the sensitivity obtained from the adapted attenuation coefficient of ^177^Lu and water (0.108 cm^−1^), an individual attenuation-related sensitivity was calculated using the law of attenuation allowing for approximation of the absolute activity-concentration in the kidneys of the torso phantom.

**Table 2:** Distances of the kidneys of the torso phantom, a 130 kg patient and a 40 kg patient to the surface of the phantom and the body respectively and the corresponding attenuation

			ventral	dorsal	left	right	absolute
Torso phantom	right kidney	Distance [cm]	9.8	6.3	17.5	8.0	
		count rate [%]	35	51	15	42	35.5
	left kidney	Distance [cm]	10.2	5.7	7.8	17.5	
		count rate [%]	33	54	43	15	36.3
Patient 130 kg	right kidney	Distance [cm]	16.1	8.4	8.6	24.5	
		count rate [%]	18	40	40	7.1	26.3
	left kidney	Distance [cm]	16.5	9.1	24.1	9.3	
		count rate [%]	17	37	7.4	37	24.6
Patient 40 kg	right kidney	Distance [cm]	10.0	3.8	4.6	19.1	
		count rate [%]	34	66	61	13	43.7
	left kidney	Distance [cm]	10.2	4.8	19.8	4.8	
		count rate [%]	33	60	12	60	41.3

In a retrospective analysis the required distances in the CT examination from the PET/CT performed in the context of therapy planning was determined for the 196 investigated patients. By using this patient-individual calibration, the remaining activity in the kidneys was calculated retrospectively in every SPECT examination of each patient to calibrate the time activity curve. Kidney doses were then estimated using the calculated activity in the kidneys and the determined effective intra-renal half-life. A mean energy deposition including gamma fraction of 2.36*10^–14^ Joule/decay was used to calculate kidney dose [6, 43]. The masses of the kidneys were received by volumetric CT analysis. The kidney dose was calculated using the Marinelli equation. The results of the calculated kidney doses were compared to data from current international literature. All mathematic calculations were performed with MS Excel 2010. Statistical analysis was performed using the designated statistic software OriginPro, Version 2020 (OriginLab Corporation, Northampton, MA, USA).

## Results

### Phantom Measurements

The kidneys of the torso phantom were filled with 41 MBq (right kidney) and 30 MBq (left kidney) respectively. The volume of the kidneys was 109 ml (right kidney) and 103 ml (left kidney) respectively. The resulting activity-concentration was therefore 0.38 MBq/ml (right kidney) and 0.29 MBq/ml (left kidney) respectively. The tomographic sensitivity of the water-filled torso phantom was calculated by measuring the distances from the kidney surface to the body surface in the four directions as shown in Fig. 1A for the right and Fig. 1B for the left kidney (Fig. 1). The resulting ratios of the count rates in each direction at the head of the gamma camera were calculated using the law of attenuation:

1 $${I}_{D}={I}_{0}*{e}^{-\mu *d}$$

using a relative intensity I_0_ of 1 in the kidney, the above-mentioned attenuation coefficient µ of 1.08 cm^−1^ and the respective distance from kidney surface to body surface in the corresponding direction d. The measured distances and resulting relative count rates are shown in (Table 2). Presuming an elliptical shape of the phantom the cumulated ratios were 0.355 for the right and 0.363 for the left kidney respectively. The sensitivity for the water filled phantom is therefore:

2 $$10.2\frac{cps}{MBq}*0.355=3.62\frac{cps}{MBq}$$

for the right kidney and.

3 $$10.2\frac{cps}{MBq}*0.363=3.70\frac{cps}{MBq}$$

for the left kidney respectively. VOI evaluation showed a recovery rate beyond 90% compared to the real activities in the phantom kidneys.

### Patient Measurements

**Fig. 2:**
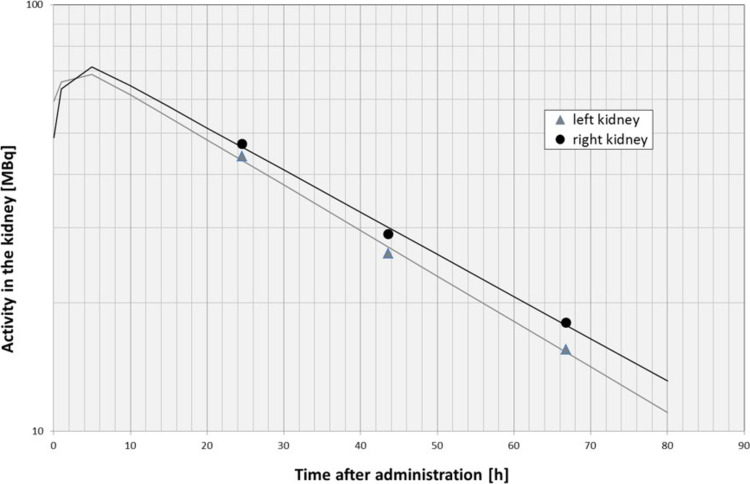
Activity in the kidneys after administration of ^177^Lu for calculation of the intra renal effective half-life in a patient (half-life of the right kidney: 30 h; half-life of the left kidney: 28 h)

The dosimetric analysis of the kidneys followed a strictly structured measuring timetable to ensure a safe and reliable treatment for each patient. Due to the highly individual bio-distribution of the radionuclide in the body, mainly depending on body weight and tumor burden, every patient received three sequential intra-therapeutic SPECT measurements (24 h, 48 h and 72 h after administration) to accomplish a reliable result of kidney dosimetry. The development of the count rate within a respective kidney VOI related to the time after administration was fitted bi-exponentially using the solver-tool of MS Excel 2010 (Fig. 2) considering an exponential influx h_2_ of the radiopharmaceutical as well as a mono-exponential phase of secretion h_1_ (Eq. 4). The influx was assumed with a half-life of one hour due to the absence of data for this time interval.

4 $$A\left(t\right)= {A}_{0 }* {e}^{-\frac{\text{ln}\left(2\right)*t}{{h}_{1}}}\times (1-{e}^{-\frac{\text{ln}\left(2\right)*t}{{h}_{2}}})$$

The fitted curve was integrated from t = 0 (time of administration) to t = ∞ (complete secretion). The gradient of the second exponential phase was used to calculate the effective intra-renal half-life. Considering the known β^−^-energy deposition per decay (E_β_) of ^177^Lu, which is 2.36*10^–14^ Joule/decay [43], and the kidney mass (m) as well as a postulated density of 1 g/cm^3^ an organ radiation dose to the kidneys was calculated following Eq. 5:

5 $$D= {A}_{0}*\frac{{E}_{\beta }}{m*\text{ln}\left(2\right)}*({h}_{1}-\frac{{h}_{1}*{h}_{2}}{{h}_{1}+{h}_{2}})$$

The absolute quantification of the activity in the kidney (A_0_) was determined by the presented patient individual sensitivity.

**Fig. 3:**
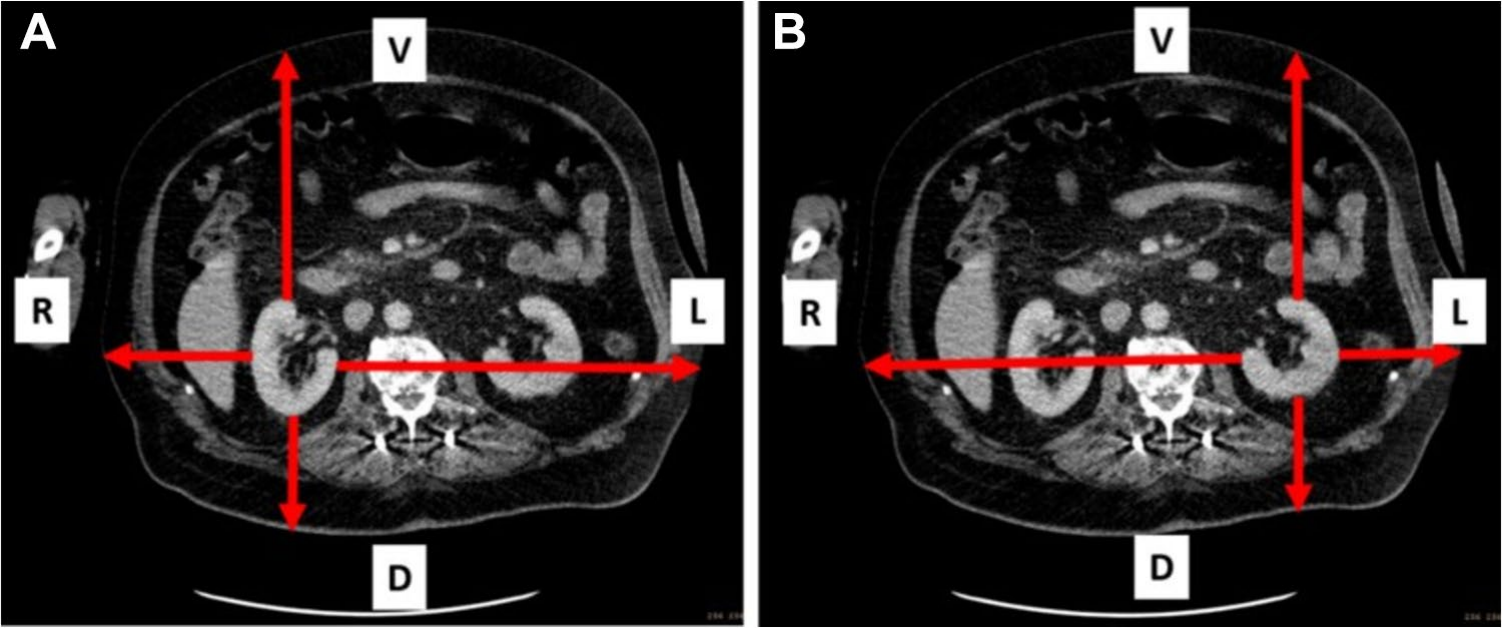
Transaxial CT-view of a 130 kg patient with measurement of the distances from the right kidney (**A**) and the left kidney (**B**) to the corresponding body surface

Example 1 describes the proposed procedure in a patient with a body weight of 130 kg. Figure 3 shows the transversal CT view of this patient (Fig. 3). The distances from the kidneys to the corresponding body surface were determined from a CT examination and are displayed in Table 2. The resulting relative amounts of the count rates are shown as well (Table 2). The individual attenuation corrected sensitivity was 2.68 cps/MBq for the right and 2.51 cps/MBq for the left kidney. With a volume of the kidneys of 224 ml (right kidney) and 245 ml (left kidney) (volumetric CT analysis) the resulting activity in the kidney 48 h after administration was 41 MBq for the right and 44 MBq for the left kidney (count rate in the kidney VOIs: 110 cps right and 111 cps left). With an effective half-life of 36 h for the right kidney and 32 h for the left kidney a radiation dose of 2.2 Gy for the right kidney and 2.7 Gy for the left kidney was calculated. This leads to an activity related radiation dose of 0.29 Gy/GBq for the right kidney and 0.36 Gy/GBq for the left kidney (administered activity: 7.54 GBq).

**Fig. 4:**
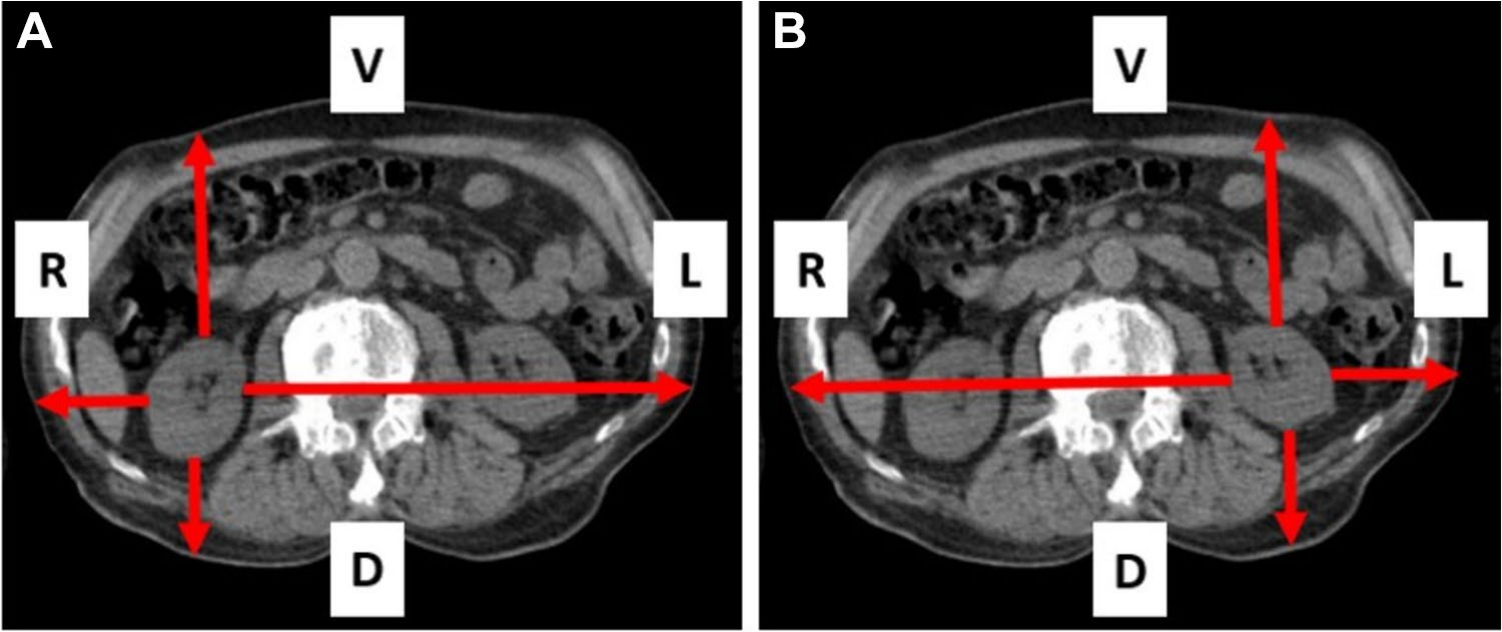
Transaxial CT-view of a 48 kg patient with measurement of the distances from the right kidney (**A**) and the left kidney (**B**) to the corresponding body surface

Example 2 shows a patient with a body weight of 48 kg (Fig. 4). The distances determined in the CT examination are displayed in Table 2. The resulting relative amounts of the count rates are shown as well (Table 2). The individual attenuation corrected sensitivity was 4.46 cps/MBq for the right and 4.21 cps/MBq for the left kidney. With a volume of the kidneys of 135 ml (right kidney) and 134 ml (left kidney) the resulting activity in the kidney 48 h after administration was 60 MBq for the right and 58 MBq for the left kidney. With an effective half-life of 30 h for the right kidney and 31 h for the left kidney a radiation dose of 5.1 Gy for the right kidney and 5.3 Gy for the left kidney was calculated. This leads to an activity related radiation dose of 1.25 Gy/GBq for the right kidney and 1.30 Gy/GBq for the left kidney (administered activity: 4.07 GBq).

### Retrospective Analysis of 1,841 Kidneys

**Fig. 5:**
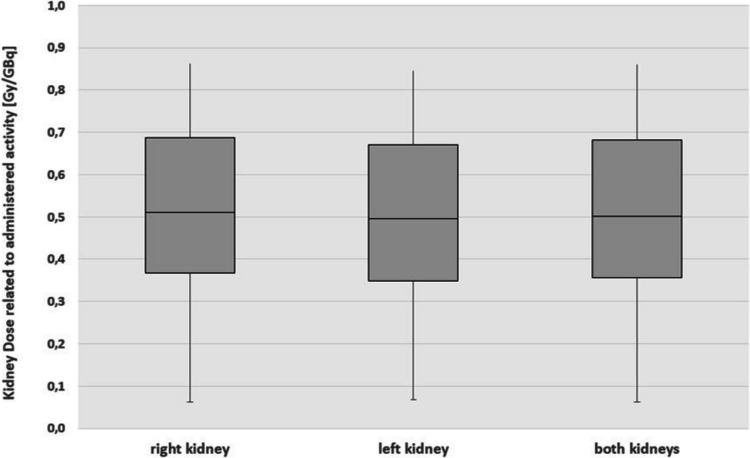
Calculated doses per administered activity for the right, the left and both kidneys

A total of 196 patients with 926 treatment cycles were included in the study. Seven patients had one non-functional kidney or unilateral nephrectomy. Therefore, 1,841 kidneys were evaluated. Mean administered activity was 7.15 ± 1.5 GBq. The mean mass of the 1,841 kidneys (917 right and 924 left kidneys) was 189 (± 41) g per kidney (Table 3). Mean effective half-life of the radionuclide in the kidney was 31.7 ± 9.8 h. The mean dose per cycle and kidney was 3.84 ± 1.87 Gy (Table 3). Mean dose per administered activity was 0.54 ± 0.26 Gy/GBq per kidney (Fig. 5; Table 3). Frequency distribution of kidney doses of the right and left kidney (Fig. 5) did not differ significantly (*p* < 0.05) applying a two-sample t-test.

**Table 3:** Dosimetric evaluation of the 1,841 kidneys

	Kidney mass [g]		Effective half-life [d]		Kidney dose [Gy]		Kidney dose [Gy/GBq]	
Kidney	Mean	Std. dev	Mean	Std. dev	Mean	Std dev	Mean	Std dev
right	185	± 41	31.9	± 9.8	3.91	± 1.91	0.55	± 0.26
left	192	± 41	31.5	± 9.7	3.78	± 1.83	0.54	± 0.26
both	189	± 41	31.7	± 9.8	3.84	± 1.87	0.54	± 0.26

## Discussion

Knowledge of kidney dose is of particular importance in ^177^Lu-PSMA RLT. The radionuclide is not only excreted by renal elimination but is also bound by specific receptors located on proximal tubule cells. Part of it remains within the cells causing an additive radiation-induced nephrotoxicity [16, 44]. Based on these findings, it is even more important to monitor the radiation dose to the kidneys during ^177^Lu-PSMA RLT. Administration of a kidney protective substance during therapy that links competitively with the receptor located in the proximal tubule to reduce renal tracer uptake is also under discussion [45, 46]. However, previous studies showed a reduced subsequent tumor uptake [46]. Therefore, this kind of nephroprotection is currently not part of daily clinical routine.

The new proposed attenuation-based dosimetry allows an absolute quantification of the kidney dose in patients undergoing ^177^Lu-PSMA RLT without the use of a SPECT/CT. Radiation doses of both the left and the right kidney did not differ significantly what indicates validity of the presented method. Assuming the dosimetrically calculated mean kidney dose of 0.54 Gy/GBq and an administered activity of 7.4 GBq per cycle, the dose to the kidneys would be 4 Gy for one treatment cycle, leading to a cumulative dose of up to 24 Gy after 6 treatment cycles. However, Mader et al. showed that extended RLT beyond the completion of standard treatment with six cycles is a feasible treatment option in selected patients with high-volume residual tumor [47]. Therefore, a monitoring process of the kidney dose for patients undergoing ^177^Lu-PSMA RLT seems mandatory. Currently, the most established process to monitor the kidney dose is a combination of SPECT and SPECT/CT imaging [48]. However, there are also other approaches for example such as serial scintigraphic whole-body images [34, 49–51]. The kidney doses calculated with the proposed method are in the same range compared to previous international studies (Table 4). Violet et al. used serial quantitative SPECT/CT at three time points after injection and achieved quantification by conversion of voxel count per second to activity per unit volume. A correction of scatter, attenuation and dead time was performed. The median kidney dose per administered activity was 0.39 (± 0.15) Gy/GBq (Table 4) [48]. The lower kidney doses per administered activity may be caused by the considerably smaller cohort of only 30 patients and differing inclusion criteria. Sarnelli et al. used anterior and posterior scintigraphic whole-body images and parameters obtained from blood samples for quantification. Mean kidney dose per administered activity was 0.67 (± 0.27) Gy/GBq (Table 4) [49]. The variance compared to the results obtained with the proposed method may be explained by the planar characteristics of scintigraphic imaging and therefore unavoidable accumulation of the counts in sagittal overlaying organs and tissue, especially in the intestines. Kratochwil et al. performed anterior and posterior scintigraphic whole-body imaging at five time points and an additional SPECT/CT to estimate the influence of overlaying intestine. Four patients were finally evaluated and the calculated mean kidney dose was 0.75 (± 0.19) Gy/GBq (Table 4) [39]. The higher kidney doses may again be explained by the low number of patients. Delker et al. acquired scintigraphic whole-body and SPECT images at four time points after administration (1, 24, 48 and 72 h p.i.). On the day of administration, an additional CT imaging was performed. Quantity was established using a 3D SPECT-OSEM reconstruction and a camera-specific correlation factor. Mean kidney dose was 0.60 (± 0.18) Gy/GBq (Table 4) [26]. The patients in this study received a mean activity of only 3.6 GBq per cycle but the kidney mass of 156 g was considerably lower than the kidney mass of the presented cohort (189 g). Rosar et al. compared the feasibility of different approaches of image-based kidney dosimetry of 24 patients [40]. The authors acquired planar whole body and SPECT/CT images at 24 h, 48 h and beyond 96 h and performed 2D planar-based dosimetry, 3D SPECT/CT dosimetry and a combination of both 2D and 3D data. 3D SPECT/CT dosimetry was again identified as the reference method and showed a mean absorbed dose of 0.54 ± 0.28 Gy/GBq which is well in line with the results obtained with the proposed method [40].

**Table 4:** Review of current literature concerning kidney dosimetry in RLT

Study	Number of patients	Mean activity per cycle [GBq]	Mean kidney dose [Gy/GBq]
Baum et al. [24]	30	5.8	0.80 ± 0.40
Kratochwil et al. [39]	18	6.0	0.75 ± 0.19
Rinscheid et al. [23]	13	7.3	0.68 ± 0.24
Sarnelli et al. [49]	9	5.0	0.67 ± 0.27
Delker et al. [26]	5	3.6	0.60 ± 0.18
Happel et al. [this study]	**196**	**7.2**	**0.54 ± 0.26**
Rosar et al. [40]	24	6.4	0.54 ± 0.28
Hohberg et al. [51]	9	5.5	0.53 ± 0.17
Violet et al. [48]	30	7.8	0.39 ± 0.15
Götz et al. [32]	13	6.9	0.39 ± 0.11

Previous studies often lack a sufficient number of patients to obtain a representative and reliable result (Table 4). Moreover, the acquisition of data by quantitative imaging requires image correction such as attenuation, dead-time, background or scatter correction that was not performed in all studies. In the present study, scatter, dead-time and background correction were used to obtain a result as accurate as possible. The calculated kidney dose of 0.54 Gy/GBq obtained with the proposed dosimetric method was within the range of the kidney doses calculated in the aforementioned studies which ranged between 0.39 and 0.80 Gy/GBq (Table 4) [39, 48].

With the new proposed method, it is possible to calculate kidney doses without the use of a SPECT/CT. However, the method has limitations. Performing scintigraphic imaging of obese patients does not achieve the usual precision due to a reduced spatial resolution which makes it challenging to draw the VOIs accurately. Due to its accuracy, kidney dose monitoring applying SPECT/CT imaging remains the preferable dosimetry procedure. Further limitations of the proposed method are an adopted homogenous density of 1 g/cm^3^ that may under or overestimate the density of the different absorbing organs and tissues. Moreover, the number of four directions is only an approach and does only fit a patient with a transversal elliptic body. The utilization of further directions would improve the accuracy of dosimetry but would also be an unwarranted time-consuming procedure. The proposed method seems to be comparable to the well-established Chang-correction that has been applied for quantification in scintigraphic images for decades [52, 53]. Chang-correction represents a mathematical approach assuming a constant attenuation coefficient in the investigated tissue and is mainly applied in scintigraphic brain imaging. It is an approximation that can only be used in tissues with a homogenous density as e.g. in the brain. The contour of the head must be approximated by an elliptically shaped region of interest, which is a reasonable assumption for the skull region [52, 53]. As a proof of concept, Chang-correction was applied in case of the investigated torso-phantom and compared to the proposed method. The results were in the same range, but it must be noted, that the torso-phantom simulates a normal weight patient. In real-world patients with a reduced or elevated body weight Chang-correction will frequently not match the real patients contour and density. Moreover, the results may highly depend on the investigated transversal slice for the drawing of regions of interest.

Another possible alternative for institutions without access to a SPECT/CT may be the calculation of the activity concentration in the kidneys using planar scintigraphic images in combination with quantitative measurements with a gamma-probe [6]. The main issue of this approach is again the unknown attenuation in the sagittal view of the patient. It is commonly accepted to use the geometric-mean of anterior and posterior views [6]. However, this is only suitable for a point-source. In the case of kidney dosimetry in radioligand patients, an unknown patient specific distribution of activity in the liver, intestine, further organs and metastases leads to an overlap of different activities in the sagittal view, making the geometric mean inaccurate for quantitative calculation.

A further issue playing an important role for quality of the performed dosimetry is the time of the CT acquisition. A long-time lag between CT examination and treatment may compromise accuracy of dosimetry due to varied dimensions of the patient. Moreover, it must be ensured that the arms of the patient are in the same posture in CT and dosimetric SPECT.

Finally, the key point of the presented data is to provide a reliable approach for quantification of intra-renal activity at the time of SPECT imaging. The following step for calculating the energy dose is calculating the total number of decays in the kidney, defined by the effective half-life of the radio-pharmakon in the kidney. The calculation of the effective half-life strongly depends on the number of measured time points and the fitting of the time activity curve. This process varies significantly in the cited references [23, 24, 26, 32, 39, 40, 48, 49, 51]. Therefore, a comparison of the energy dose to the kidney as performed in the presented study has a certain potential for error. However, as none of the compared studies from Table 4 [23, 24, 26, 32, 39, 40, 48, 49, 51] indicated the absolute activity in the kidney, the only possible way to compare the data was by comparison of the calculated energy doses to the kidneys. Therefore, the results should be viewed critically in this regard.

## Conclusions

The new proposed attenuation-based procedure provides a path to quantify kidney doses in patients undergoing ^177^Lu-PSMA RLT without SPECT/CT. The obtained results were validated with several common approaches and are comparable to previous methods of kidney dosimetry from the literature. The wide range of the reported kidney doses obtained with different methods mark the requirement to implement a regular and established individual procedure of kidney dosimetry.

## Data Availability

All data are available from the corresponding author.
